# E-mental health implementation in inpatient care: Exploring its potential and future challenges

**DOI:** 10.3389/fdgth.2022.1027864

**Published:** 2022-12-14

**Authors:** Eva Van Assche, Bert Bonroy, Marc Mertens, Lore Van den Broeck, Kimberly Desie, Felix Bolinski, Khadicha Amarti, Annet Kleiboer, Heleen Riper, Tom Van Daele

**Affiliations:** ^1^ Thomas More University of Applied Sciences, Antwerp, Belgium; ^2^ Thomas More University of Applied Sciences, Geel, Belgium; ^3^ Pulso Europe, Leuven, Belgium; ^4^ Department of Clinical, Neuro, and Developmental Psychology, Amsterdam Public Health Institute, Vrije Universiteit Amsterdam, Amsterdam, Netherlands; ^5^ Department of Psychiatry, Amsterdam University Medical Centre (VUmc), Amsterdam, Netherlands; ^6^ Faculty of Medicine, University of Turku, Turku, Finland

**Keywords:** e-mental health, psychiatric inpatient care, implementation, promoting and hindering factors, depression

## Abstract

**Background:**

There is a great evidence base today for the effectiveness of e-mental health, or the use of technology in mental healthcare. However, large-scale implementation in mental healthcare organisations is lacking, especially in inpatient specialized mental healthcare settings.

**Aim:**

The current study aimed to gain insights into the factors that promote or hinder the implementation of e-mental health applications on organisational, professional and patient levels in Belgium.

**Methods:**

Four Belgian psychiatric hospitals and psychiatric departments of general hospitals invited their professionals and patients to use Moodbuster, which is a modular web-based platform with a connected smartphone application for monitoring. The platform was used in addition to treatment as usual for three to four months. The professionals and patients completed pre- and post-implementation questionnaires on their reasons to participate or to decline participation and experiences with the Moodbuster platform.

**Results:**

Main reasons for the organisations to participate in the implementation study were a general interest in e-mental health and seeing it is a helpful add-on to regular treatment. The actual use of Moodbuster by professionals and patients proved to be challenging with only 10 professionals and 24 patients participating. Implementation was hindered by technical difficulties and inpatient care specific factors such as lack of structural facilities to use e-mental health and patient-specific factors. Professionals saw value in using e-mental health applications for bridging the transition from inpatient to outpatient care. Twenty-two professionals and 31 patients completed the questionnaire on reasons not to participate. For the patients, lack of motivation because of too severe depressive symptoms was the most important reason not to participate. For professionals, it was lack of time and high workload.

**Conclusions:**

The current implementation study reveals several important barriers to overcome in order to successfully implement e-mental health in inpatient psychiatric care.

## Introduction

The potential of technology for mental health support and mental healthcare has been the focus of research for over two decades. Synchronous and asynchronous communication, immersive technologies and wearables, and especially digital (self-help) interventions, also called e-mental health interventions, have been developed and thoroughly evaluated in controlled settings, often with favourable results (1–3). Although the demand for mental health support and psychotherapy continues to increase, the routine implementation of technology remains largely absent and is often insufficiently considered as a viable option to improve mental healthcare (4–6).

It took a pandemic for a swift and massive adoption of technology, in which mental healthcare professionals made a (temporary) transition from face-to-face to online consultations, often through videoconferencing software, to be able to continue their services (7, 8). However, online consultations are only one aspect of technology in mental healthcare and offer limited resolve regarding ongoing issues in mental healthcare systems around the world. Major challenges remain such as high demand and difficult remote services (4, 9). These challenges are even expected to increase, as the demand for mental healthcare has been on the rise and is expected to further increase over the next years (10). One of the opportunities to increase access to, and potentially also the efficiency of, care is by implementing e-mental health interventions more robustly in routine health care settings. This means that online consultations can remain as a means for remote delivery, whereas digital (self-help) interventions can be implemented to reduce waiting lists or even as high-quality stand-alone care for mild to moderate symptoms (11).

In parallel, e-mental health interventions are being implemented as an addition to conventional care, or in combination with conventional care, which is often referred to as “blended care” (12–14). Blended care integrates online and face-to-face interventions into one treatment protocol (15) and is considered by both mental health care professionals and patients as a more acceptable way to approach the use of technology in psychotherapy (16). However, the evidence-base for blended care is not as extensive compared to digital interventions (2, 11, 12).

Furthermore, positive results from clinical trials in terms of achieved effectiveness and uptake, even when embedded in clinical practice, seem to contrast with the more mixed results in real-life settings (11, 17). Research on the translation of these clinical trial results into routine care is scarce (5), and the uptake of and adherence to e-mental health interventions outside of research trials is often poor, both for professionals and patients (18). The need for implementation research focussing on embedding and integrating new interventions in routine care settings is high. Vis et al. (5) therefore identified barriers and facilitating factors relevant to the implementation of e-mental health for mood disorders. An important barrier relates to the expectations and preferences of professionals and patients about e-mental health interventions. Negative attitudes and expectations often lead to unsuccessful implementation of e-mental health. Another important determinant for successful implementation was the appropriateness of the e-mental health intervention in addressing the mental health disorder. Finally, the availability, stability and reliability of the e-mental health intervention are important determinants for its long-term use.

Importantly, the focus of implementation studies for e-mental health interventions has been mainly on outpatient care only (19, 20) and results on implementation in psychiatric inpatient settings are missing. There are, however, a limited number of studies focusing on e-mental health interventions within German psychiatric inpatient care. Zwerenz et al. (21) evaluated the acceptance and efficacy of an online self-help CBT program Deprexis as an add-on to the treatment of depression in German inpatient psychodynamic settings. They included an intervention group that used Deprexis and an active control group that received weekly online information on depression. After having access to their respective interventions for 12 weeks, depressive symptoms were lower in the intervention group compared to the active control group. Even though professionals initially were concerned the online intervention would overburden depressed patients in these settings, these patients showed stronger and more lasting treatment effects (22). These promising results suggest that e-mental health interventions as an add-on to regular treatment in inpatient settings are effective, but the authors also highlight future challenges related to thoughtful and sustainable implementation and reimbursement issues.

In another German study, Dorow et al. (23) investigated the acceptance, chances and barriers of an online self-management CBT program for depression called Moodgym. Patients used Moodgym autonomously for 8 weeks as an addition to regular treatment and reported on their experiences in a pre-post assessment with written questionnaires. They reported moderate to high user acceptance, but professionals also reported barriers such as limited technological skills, concentration problems and severe course of depression for patients. They nevertheless concluded that the online program could serve as an additional treatment option in inpatient care.

Similarly, Sander et al. (24) identified potential benefits, facilitators and barriers for the implementation of digital interventions in a study with professionals working in inpatient care. They also assessed the attitude towards digital interventions, regardless of level of professionals guidance that is involved. Professionals of different psychiatric hospitals participated through on site workshops. They were first given access to the online therapy program Moodbuster (https://www.moodbuster.science), to make sure they all had experience with a digital therapy program before completing a questionnaire on their attitudes, benefits, and perceived facilitators and barriers in the workshop. Results showed professionals' attitudes were rather neutral. Most important benefits included optimised treatment structure and extension of treatment spectrum because e-mental health applications can serve as helpful add-ons to inpatient regular therapy. Facilitators for the implementation of digital interventions in inpatient care identified in Sander et al. could be at a technical, patient-related and organisational level. The most important facilitators were respectively high usability of the e-mental health intervention, sufficient cognitive and functional ability of patients to use the intervention, and sufficient training for professionals. Possible barriers for use of e-mental health in inpatient care were lack of necessary cognitive capabilities in patients because of too severe symptoms, insufficient technical equipment and lack of internet access. Indeed, when exploring potential causes for the reluctant uptake of blended therapy, there is a wide variety of factors that cannot only facilitate, but also impede implementation success. In general, these factors can be situated at the policy level (25, 26), at the level of mental healthcare professionals (27, 28), and at the patient level (29).

Research to date has primarily focused on each level individually but has not taken on a broader perspective by all three levels at the same time, and especially not in inpatient care. The objective of this Belgian study was therefore to gain insight into factors that promote or hinder implementation of e-mental health applications in inpatient specialised mental healthcare organisations by assessing the viewpoints of three actors in the implementation process: (1) mental healthcare organisations, (2) mental healthcare professionals and (3) patients. In Belgium, e-mental health is still underrepresented at policy and practice levels but awareness is increasing. A lack of regulations and a complex state structure in which mental healthcare responsibility has been de-federalised are partly responsible for the slow implementation of e-mental health into routine care settings (4). We first explored perspectives on e-mental health in inpatient psychiatric care from an organisational level. In addition, we investigated their willingness to participate in the actual implementation study in which Moodbuster, an online cognitive behavioural therapy program, was used as an add-on to treatment as usual (TAU). Moodbuster was implemented in four inpatient (psychiatric) hospitals who expressed their interest in the study. We captured the actual uptake by the professionals and patients and their implementation experiences.

## Materials and methods

### Moodbuster

This study was conducted in the context of the European project eMEN, which aims to increase knowledge on the implementation of e-mental health applications in Europe and was funded by the Interreg North-West Europe Innovation Program. Moodbuster (https://www.moodbuster.science/) was selected as the e-mental health application because it is a research platform - non-commercial - for the online treatment of depression and is available in several languages such as Dutch, English, French, German, and the Belgian variant of Dutch, Flemish, which was developed for the current study. It has been developed by Vrije Universiteit (VU) Amsterdam and INESC TEC within the ICT4Depression project (30). Clinical effectiveness and cost-effectiveness of blended treatment of major depression with Moodbuster compared to regular treatment was evaluated within the E-COMPARED project (15). Kemmeren et al. (31) showed in studies in Germany, the Netherlands, Poland and France that blended treatment with Moodbuster can be applied to patients with depression in routine care and that patients who did not comply with the allocated blended treatment often had more comorbidities. Moodbuster can be used for both prevention and treatment, as a self-help tool, in guided online treatment or in blended treatment, and in outpatient and inpatient settings (15). It offers cognitive behavioural therapy for depression, which consisted of two mandatory modules: an introduction (explaining the program and how to use it) and a psychoeducation module. Upon completion of these modules, four optional treatment modules were offered in which the patient learned how to: think more positively, plan enjoyable activities, apply different ways of problem solving and be physically active. A final module was offered for relapse prevention. The platform consists of a web portal for patients and for mental healthcare professionals. In addition, patients can make use of a connected mobile app to monitor variables such as mood, sleep or daily activities.

In the current study, we opted for a pragmatic implementation of Moodbuster as professionals were allowed to use this e-mental health platform in addition to TAU, meaning that they offered it as a supplement to regular therapy as they saw fit in their specific psychiatric and organisational settings. Therefore, no treatment protocol was provided, specifying when, how and in which manner the platform should be integrated within their routine care. The specific conditions in and heterogeneity of inpatient mental healthcare settings did not allow otherwise. This contrasts with controlled studies using blended treatment protocols for actual use and progress of the e-mental health intervention, often specifying number, timing and content of e-mental health sessions and face-to-face sessions (19). However, Moodbuster is a flexible platform offering the option to tailor treatment to individual patients. Indeed, in Kemmeren et al. (31), blended treatment protocols for depression with Moodbuster were often not followed exactly as intended, with therapists and patients personalizing the blended care approach.

### Recruitment of mental healthcare organisations, professionals and patients

All 66 Flemish psychiatric hospitals and psychiatric units of general hospitals in Belgium were invited for participation in the current implementation study on the use of an e-mental health application for depression through e-mail and phone calls. Responsible members of staff of each hospital could indicate their interest in the study and four were selected for the actual implementation of Moodbuster. We selected hospitals based on motivation for participation, geographical accessibility, and diversity in the therapeutic context. One hospital started the implementation in September 2019, whereas the three other started in November 2019 because lack of time, and one hospital just went through a re-organisation process in the preceding months. The implementation period lasted for three months, but two hospitals requested to use Moodbuster one additional month to allow for more time to include patients. Ethical approval for this study was obtained from the central ethics committee of the GZA hospitals Antwerp (GZA 190504ACADEM) and the local ethics committees of the other participating hospitals (Jessa hospital, Sint-Franciscus hospital, University Psychiatric Centre Duffel).

Information sessions for the mental healthcare professionals were organized in each of the participating hospitals to inform them about the purpose and setup of the study and give them a demonstration of the Moodbuster platform. This was done in cooperation with the Moodbuster platform development team of the VU Amsterdam. Any mental healthcare professional who had a mandate for regular therapeutic contact with patients having depression or depressive symptoms was eligible for participation.

After each information session, professionals could make an informed decision whether or not to participate in the implementation study. Professionals willing to participate invited eligible patients and told them about the setup and goals of the study. We also provided an information folder for patients to provide them with the necessary information to make an informed decision. We followed-up on the Moodbuster implementation by regular e-mail contact with the professionals and site visits. In case professionals experienced problems with or had questions about the Moodbuster platform and or the app, they could contact the researchers of Thomas More University of Applied Sciences, who were in close contact with the Moodbuster platform development team.

### Materials

#### Organisations

Hospitals who indicated their interest in the study were invited to complete an online survey asking first for the name of the organisation and function of the person completing it, followed by two open-ended questions about the organisation's previous experience with e-mental health applications and reasons for participation.

#### Professionals

Professionals and patients completed questionnaires both at the beginning and end of the implementation period which was three months in two hospitals and four months in the other two (psychiatric) hospitals.

Professionals who agreed to participate after the information session received a questionnaire with open-ended questions on socio-demographics (age, professional function, gender, years of professional experience), their previous experience with e-mental health applications and reasons for participation.

At the end of the implementation period, professionals responded to open-ended questions on their experiences with Moodbuster, a questionnaire based on the Client Change Interview (CCI 32);, and two validated questionnaires to measure satisfaction with the intervention and perceived usability. The Client Satisfaction Questionnaire (CSQ-3 33); consists of the three most salient items to measure perceived satisfaction with a service, in this case the use of Moodbuster as an add-on to TAU, and has four response options. The System Usability Scale (SUS 34); assesses the usability of a wide variety of products and services and has ten questions with five response options. In this study, it measured perceived usability of the Moodbuster platform in professinals' daily work.

Mental healthcare professionals who did not want to enrol, were asked to complete a short questionnaire on socio-demographics, previous experience with e-mental health applications and reasons for refusal.

#### Patients

Eligible patients of the respective participating professionals also completed a questionnaire on socio-demographics, their previous experiences with e-mental health applications and reasons for participation, before they started using the Moodbuster platform.

At the end of the implementation period, participants were asked to complete the eight item Client Satisfaction Questionnaire, and the SUS, a questionnaire based on the CCI and a question on their evaluation of and experiences with the Moodbuster tool.

Similar to the procedure for professionals, non-participating patients were asked to complete a questionnaire on socio-demographics, previous experience with e-mental health applications and reasons for refusal.

### Analysis

Quantitative descriptive analysis providing mean values (*M*) and standard deviations (*SD*) was performed for age, years of professional experience, CSQ and SUS. An inductive approach (thematic analysis) was used for the qualitative analysis of the responses to the open questions on (1) reasons for participation for the organisations, professionals and patients, (2) experiences with the Moodbuster platform of professionals and patients, (3) reasons for declining participation of professionals and patients. Responses were paraphrased and a list of response themes was developed according to which each response was coded. Frequencies of each theme were calculated and the most frequent ones were reported in the results. Coding and analyses were performed by the first author.

## Results

**Figure 1:**
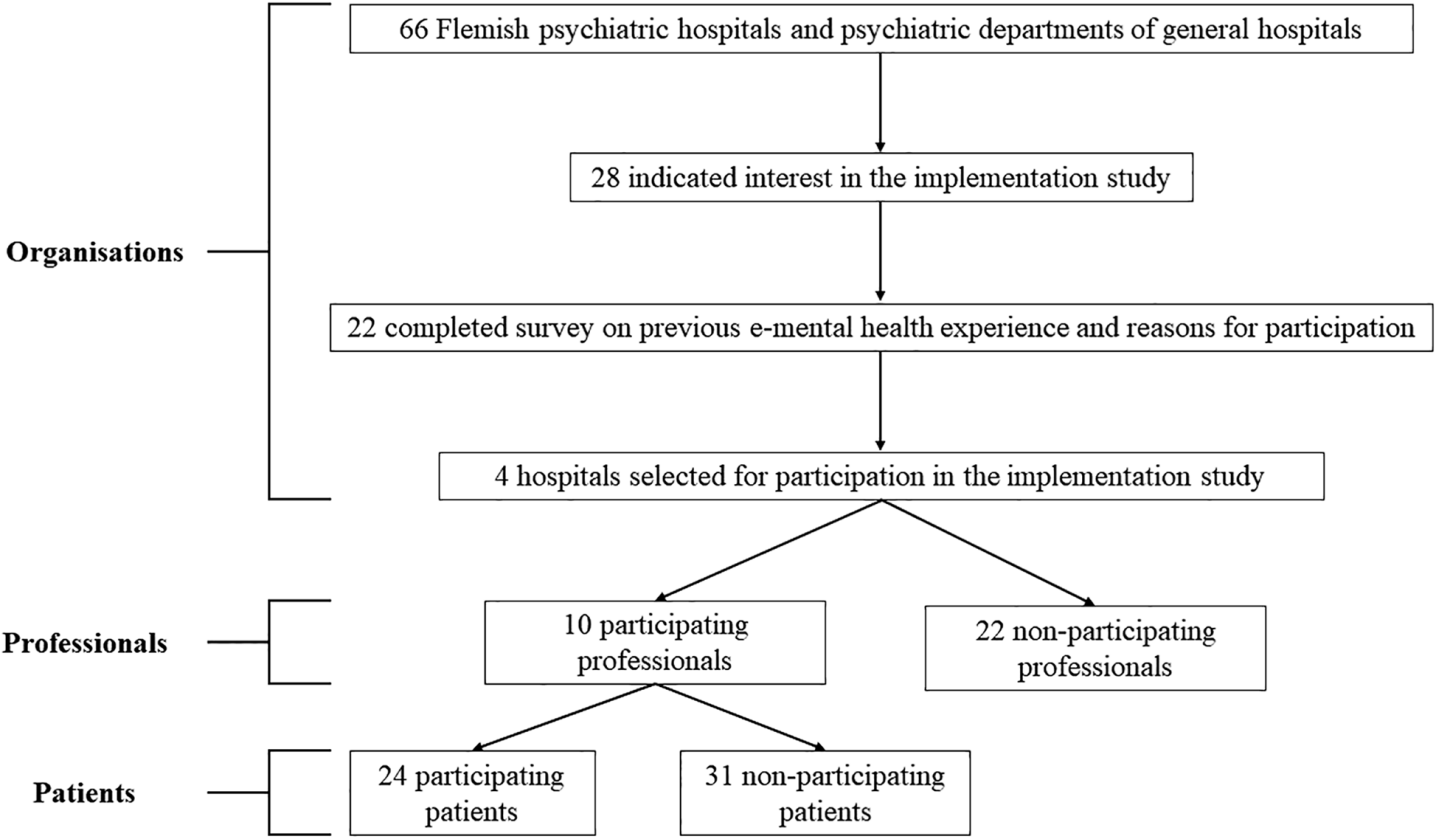
Participant flow diagram.

The recruitment and participation flow is presented in Figure 1.

### Organisations

Twenty-eight (psychiatric) hospitals indicated their interest in the study and 22 responsible members of staff from each hospital completed the online survey (*N* = 22). Fourteen of them indicated that their organisation had no previous experience with e-mental health applications, whereas eight reported having previous experience. This included the use of digital psychoeducation, online diagnostics and questionnaires, and the use of e-mental health applications. The most important reasons for participation in the implementation study were related to: extra possibilities in addition to regular treatment (*n* = 8/22), general interest in e-mental health possibilities (*n* = 8/22), gain experience in using technological applications (*n* = 4/22), provide better continuity of care after residential care (*n* = 3/22), and increase patient participation (*n* = 3/22). The actual implementation of Moodbuster took place in 4 of the 22 (psychiatric) hospitals that indicated they were interested in participating in the implementation study.

### Participating professionals

There were 10 participating professionals of which eight were female and two were male. Four of them were psychologists, three were psychology interns, and three were (psychiatric) nurses. Age ranged between 23 and 52, with a mean of 33.10 (*SD* = 10.13). They had on average 9.50 years of professional experience (range 0 to 30; *SD* = 10.28).

The average score on the SUS (10 items; α = 0.33) was 48.13 (range 40–63; *SD* = 9.52). This indicates a score between the range of poor to OK, but unacceptable for the user-friendliness of the Moodbuster platform (35). The client satisfaction with the Moodbuster platform measured with the CSQ-3 (3 items; *α* = 0.75) was rather low with a mean of 5.56 out of 12.

Results showed that six professionals had no previous experience with using technological applications. Two had experience with using apps and websites for suicide prevention, alcohol disorders and relaxation exercises. The remaining two professionals did not provide information on this. Their reasons for participating in the implementation study were mainly that they see e-mental health applications as an important addition to regular treatment (*n* = 4/10), they want to gain experience with it (*n* = 3/10), and they believe it will become increasingly important in the future (*n* = 3/10). However, results of the post-implementation questionnaire revealed several barriers for the implementation of Moodbuster. Technical difficulties with the mobile app (*n* = 2/10) and lack of structural facilities in the hospital (such as good internet connection and access to pc or tablet) (*n* = 4/10) made it difficult to fully use the potential of Moodbuster. Other barriers were the fact that the use of Moodbuster was often not supported by the whole professional team (*n* = 2/10) and the lack of time to use Moodbuster in addition to regular therapy (*n* = 2/10). There was also a lack of interest in patients (*n* = 2/10) and the short stay in the hospital (typically three to five weeks) made it difficult for patients to become familiar with the platform. Professionals do see potential in using Moodbuster for longer patient trajectories also involving outpatient care.

### Non-participating professionals

Twenty-two non-participating professionals completed the questionnaire on socio-demographics and reasons for refusal. They were on average 39 years old (range 21–58; *SD* = 12.04). Four of them were male, 17 female and one professional did not report gender. Their professional function was (psychiatric) nurse (*N* = 15), psychologist (*N* = 2), therapist (*N* = 2), and three did not complete professional information. The answers on reasons for refusal showed that lack of time (*n* = 12/22) and high workload (*n* = 11/22) were the main reasons for declining participation. Other reasons mentioned were staff shortage (*n* = 4/22), insufficient information (*n* = 4/22), and doubt that patients will understand and be able to use the Moodbuster platform (*n* = 3/22).

### Participating patients

The 24 patients who participated in the implementation study were on average 43 years old (range 26–66; *SD* = 9.98). Eleven of them were male, 13 were female. The majority (*N* = 17) was on sick leave, two were unemployed, three were employed, one was unemployed because of permanent disability and one patient was retired. Ten had a degree of higher education, 11 of secondary education and three had a primary school degree.

After the Moodbuster implementation period, only eight patients completed the post-implementation questionnaire with the CSQ-8 (8 items; *α* = 0.81) and the SUS (10 items; *α = *0.84). The average score of 17.33 (range 9–22; *SD* = 4.63) out of 32 on the CSQ-8 was sufficient for the client's satisfaction with the tool. The mean score of 55.71 (range 35–80; *SD* = 14.27) out of 100 on the SUS was also sufficient for the usability of the tool.

Analyses showed that the majority had no previous experience with e-mental health applications (*N* = 18/24). Only three reported having experience and three did not complete this question. The most frequent reasons for participation were: to use the Moodbuster platform as an extra help in treatment (*n* = 12/24), to gain experience with e-mental health (*N* = 5/24), and to help scientific research (*n* = 3/24). Similar to the professionals, they also experienced technical problems with the mobile application (such as difficulties installing the app and push notifications for mood rating) (*n* = 6/24). Patients did see value in the platform and especially the mood registration and follow-up (*n* = 4/24).

### Non-participating patients

Thirty-one patients who declined participation completed the questionnaire on their reasons for decline. Their mean age was 48.07 years (range 18–75; *SD* = 16,76). Seventeen were female, 13 were male and one person did not complete this question. Most of them had a degree of secondary education (*n* = 20/31), the others had either a degree of higher education (*n* = 7/31) or primary education (*n* = 3/31), or did not provide an answer (*n* = 1/31). Twenty had no previous experience with e-mental health applications, whereas 6 had experience with using apps and chat. The most important reasons for declining participation were lack of motivation because of severe depressive symptoms (*n* = 7/31), lack of interest (*n* = 6/31), being not open to use of technology in mental health care (*n* = 5/31), limited knowledge of technology (*n* = 4/31) and the fact that no technological hardware was available (*n* = 3/31).

## Discussion

This pragmatic implementation of the e-mental health application Moodbuster in Belgian inpatient care provided first insights into the perspectives on and experiences with e-mental health on organisational, professional, and patient levels. The majority of (psychiatric) hospitals who expressed their interest in implementing Moodbuster had no previous experience with e-mental health and were in doubt whether there was sufficient time for the implementation of e-mental health. Main reasons for participating in the implementation study were a general interest in e-mental health and seeing it as a helpful add-on to regular treatment. This last reason was also seen by German professionals in inpatient care as an important benefit of e-mental health (24). Indeed, European organisations are aware of the potential benefits of e-mental health interventions but differ in terms of level of knowledge about these interventions and their feasibility within routine care. According to Topooco et al. (36), it is considered by therapists to be more suitable for milder forms of depression and in the context of blended care.

Even though the hospital's responsible agreed to participate in the study, the actual implementation of Moodbuster in four (psychiatric) hospitals proved challenging as in total only 10 professionals participated. Implementation was hindered by technical difficulties with the app and inpatient care specific factors such as lack of structural facilities to use e-mental health. Patients experienced the same difficulties as the professionals during the Moodbuster implementation. Professionals did see potential for Moodbuster as bridging the transition from inpatient to outpatient care, as did patients who mentioned on top of it the value of mood registration in the app. Patients also in general mentioned the app for mood ratings more than the Moodbuster platform including the different modules in their questionnaire responses. This probably relates to the lack of a digital infrastructure in the inpatient hospitals. One can imagine that it is indeed easier to use Moodbuster on a smartphone compared to having to rely on a tablet or computer for this. Reasons for professionals to decline participation related to lack of time and the high workload already experienced, whereas the most important reason for patients was lack of motivation because of too severe depressive symptoms.

It thus seems that various factors contributed to the poor implementation results. One important factor possibly relates to the Belgian context where mental health care organisations have little experience with e-mental health (4), especially inpatient care settings. Also, patients admitted to these hospitals are going through a crisis period and the short stay of three to five weeks makes it difficult to get used to a new e-mental health intervention. Even though Zwerenz et al. (21) showed that an e-mental health intervention as add-on to regular treatment was implemented successfully in inpatient care, Dorow et al. (23) reported similar barriers such as concentration problems and severe course of depression for patients. The present implementation results also resemble the results of Sander et al. (24) where benefits, barriers and facilitators for implementation and attitudes of mental healthcare professionals were investigated, but no specific e-mental health intervention was implemented. They reported the importance of high usability of the intervention, sufficient ability of the patients to use it, sufficient training of professionals, and sufficient hardware and internet access. These last two conditions were not fulfilled in the present study: professionals could use the Moodbuster platform as they saw fit and it was not carried by the whole team. In addition, sufficient hardware was lacking for patients.

Results should be carefully interpreted because of small sample sizes. Nevertheless, we believe the small sample sizes are indicative of the difficulties inpatient care organisations and professionals face when implementing e-mental health and are in agreement with the low uptake and adherence to e-mental health interventions outside of research trials (18). Organisations of the current study expressed their interest in implementing an e-mental health application for depression, but an actual implementation strategy carried by the whole team was missing, leading to fragmented use of Moodbuster. Indeed, because of the specific inpatient settings and heterogeneity between these settings, professionals were given freedom to use Moodbuster as add-on to regular therapy. No structured implementation protocol was used. That is, the mental health organisations did not offer specific technical infrastructure or e-mental health training and the few therapists that used Moodbuster did it on their own initiative. This probably reflects reality in practice, but emphasizes the need for structured implementation in order to benefit from e-mental health. More controlled studies with treatment protocols stating minimum number of online sessions and recommended time frame to progress through the Moodbuster modules may have the advantage of providing more structure for the professionals to better implement e-mental health (31).

We should also note that the present data were collected right before the corona pandemic started in 2020, even leading to difficulties in collecting the completed questionnaires from the hospitals. Many mental health professionals in Belgium gained experience with online consultations during the pandemic (7) and this could lead to increased willingness to use e-mental health as an add-on or in a blended format to manage an increasing demand for mental health services (37, 38).

The current implementation study nevertheless reveals several important barriers to overcome in order to successfully implement e-mental health in inpatient psychiatric care. Although the research component involved here was kept limited, it still not fully reflects “naturalistic” implementation and usage in clinical practice, but nevertheless does approach it. These study results may therefore provide some counterweight to overly optimistic statements and expectancies regarding the potential of technology within mental healthcare, and specifically inpatient care, a sentiment increasingly shared when real-life data on e-mental health usage is being scrutinized (39, 40). It shows the complex interplay of a wide range of factors at different levels: e.g., the importance of technical infrastructure to be able to use an e-mental health application, the optimisation of usability of applications for both professionals and importantly patients who because of the severity of their illness may not be mentally able to use the application, and the time needed for professionals to learn how to incorporate e-mental health as a part of their routine care. To gain further knowledge on implementation of e-mental health in the specific setting of inpatient care, future studies should take into account the present barriers and use a broad view on implementation research, taking into account the organisational, professional and patient levels.

## Data Availability

The raw data supporting the conclusions of this article are available from the first author, Eva Van Assche, eva.vanassche@thomasmore.be.
